# Editorial: Updates and discussions about basal ganglia and their circuits

**DOI:** 10.3389/fnana.2026.1888750

**Published:** 2026-06-18

**Authors:** José Rodolfo Lopes de Paiva Cavalcanti, Expedito Silva do Nascimento-Júnior, Zhi-Li Huang, Adhil Bhagwandin

**Affiliations:** 1 Laboratory of Experimental Neurology, Department of Biomedical Sciences, State University of Rio Grande do Norte, Mossoró, Brazil; 2 Laboratory of Neuroanatomy, Department of Morphology, Biosciences Center, Federal University of Rio Grande do Norte, Natal, Brazil; 3 Department of Pharmacology, School of Basic Medical Sciences, Fudan University, Shanghai, China; 4 Division of Clinical Anatomy and Biological Anthropology, Department of Human Biology, Faculty of Health Sciences, University of Cape Town, Cape Town, South Africa

**Keywords:** basal ganglia, circuits, direct pathway, dopamine, hyperdirect pathway, indirect pathway

The basal ganglia (BG) comprise a complex network of interconnected subcortical nuclei traditionally including the striatum (caudate nucleus, putamen, and nucleus accumbens), globus pallidus (internal and external), subthalamic nucleus, and substantia nigra (*pars* compacta and *pars* reticulata) (Lanciego et al., 2012; Calabresi et al., 2014). Although historically associated with motor control, these regions are now recognized as participating in a broad range of functions involving cognition, motivation, reward processing, and emotional regulation (Graybiel, 2000; Obeso et al., 2000).

In this context, this canonical model, together with the extensive dopaminergic, glutamatergic, and GABAergic connectivity of the basal ganglia, still provides an important basis for understanding their functional diversity. In particular, the nigrostriatal, mesolimbic, and mesocortical dopaminergic pathways originating mainly from the substantia nigra pars compacta and ventral tegmental area, remain central to our understanding of neuropathological disorders such as Parkinson's and Huntington's diseases (Björklund and Dunnett, 2007).

As previously discussed, by Gerfen (2023), the classical models of BG organization proposed that the direct and indirect pathways exert opposing influences over movement, with the former facilitating and the latter suppressing motor actions, respectively. In parallel, the hyperdirect pathway, characterized by excitatory cortical projections to the subthalamic nucleus, reinforces inhibitory basal ganglia output and further contributes to movement regulation (Nelson and Kreitzer, 2014).

In fact, this framework remains highly influential from both conceptual and didactic perspectives. However, recent studies have demonstrated concurrent activation of both pathways during movement initiation and execution, challenging the traditional “go/no-go” interpretation of basal ganglia function. Additionally, recent findings indicate that non-canonical striosomal direct and indirect pathways may modulate dopamine release and motor behavior in ways that extend beyond the traditional matrix-based circuitry, adding another layer of complexity to current models of basal ganglia function (Cui et al., 2013; Varin et al., 2023; Rocha et al., 2026).

In this Research Topic, the submitted manuscripts explore multiple dimensions of basal ganglia research, ranging from classical neuroanatomical organization to contemporary imaging and therapeutic perspectives. Together, these studies reinforce the steadfast relevance of basal ganglia investigations for both basic and translational neuroscience.

A solid conceptual foundation was laid by Rocha et al., whose mini-review addresses the functional anatomy, neurochemistry, and circuit dynamics of the basal ganglia, with particular focus on the direct and indirect pathways and their dopaminergic modulation in voluntary movement. This timely review serves as an introduction for the more specialized works that follow.

The dopaminergic system was revisited here from two complementary angles. Guissoni Campos et al. investigated the expression of clock proteins Per1, Per2, and Cry1 in the substantia nigra and subthalamic nucleus of *Sapajus apella*, a diurnal primate, and concluded that these circadian regulators are present in key motor areas of the basal ganglia. Conversely, Azambuja et al. examined whether normal aging causes detectable loss of TH-immunopositive neurons or volumetric changes in the substantia nigra pars compacta and ventral tegmental area of the common marmoset.

Striatal vulnerability under chronic stress was the focus of Lira-Bandeira et al., who used stereological methods to evaluate the effects of social isolation and ayahuasca treatment on striatal neurons in juvenile marmosets. Furthermore, two contributions examined basal ganglia pathology through neuroimaging. Wang et al. explored the relationship between enlarged perivascular spaces in the basal ganglia and cerebral perfusion in elderly patients, while Zuo et al. used resting-state fMRI to assess cerebellar-cerebral connectivity in patients with cognitive impairment after basal ganglia stroke.

The final two articles addressed therapeutic possibilities. Singh and Reynolds reviewed the application of focused ultrasound to basal ganglia disorders, covering mechanisms ranging from thermal ablation and blood-brain barrier opening to neuromodulation and sonogenetics, with a discussion of early clinical evidence in Parkinson's disease, essential tremor, and dystonia. Lucena et al. took a different approach, systematically reviewing the neuroprotective effects of marine algae-derived polysaccharides in animal models of basal ganglia neurodegeneration.

Collectively, these articles demonstrate progress of basal ganglia research, from cellular morphology and circadian biology to functional neuroimaging and therapeutic innovation. We believe this assemblage of studies will be useful to both researchers approaching this complex topic for the first time and those already contributing to the field.

## References

[B1] Björklund A. Dunnett S. B. (2007). Dopamine neuron systems in the brain: an update. Trends Neurosci. 30, 194–202. doi: 10.1016/j.tins.2007.03.006 17408759

[B2] Calabresi P. Picconi B. Tozzi A. Ghiglieri V. Di Filippo M. (2014). Direct and indirect pathways of basal ganglia: a critical reappraisal. Nat. Neurosci. 17, 1022–1030. doi: 10.1038/nn.3743 25065439

[B3] Cui G. Jun S. B. Jin X. Pham M. D. Vogel S. S. Lovinger D. M. . (2013). Concurrent activation of striatal direct and indirect pathways during action initiation. Nature 494, 238–242. doi: 10.1038/nature11846 23354054 PMC4039389

[B4] Gerfen C. R. (2023). Segregation of D1 and D2 dopamine receptors in the striatal direct and indirect pathways: an historical perspective. Front. Synaptic Neurosci. 14:1002960. doi: 10.3389/fnsyn.2022.1002960 36741471 PMC9892636

[B5] Graybiel A. M. (2000). The basal ganglia. Curr. Biol. 10, R509–R511. doi: 10.1016/S0960-9822(00)00593-5 10899013

[B6] Lanciego J. L. Luquin N. Obeso J. A. (2012). Functional neuroanatomy of the basal ganglia. Cold Spring Harb. Perspect. Med. 2:a009621. doi: 10.1101/cshperspect.a009621 23071379 PMC3543080

[B7] Nelson A. B. Kreitzer A. C. (2014). Reassessing models of basal ganglia function and dysfunction. Annu. Rev. Neurosci. 37, 117–135. doi: 10.1146/annurev-neuro-071013-013916 25032493 PMC4416475

[B8] Obeso J. A. Rodríguez-Oroz M. C. Rodríguez M. Lanciego J. L. Artieda J. Gonzalo N. . (2000). Pathophysiology of the basal ganglia in Parkinson's disease. Trends Neurosci. 23, S8–S19. doi: 10.1016/S1471-1931(00)00028-8 11052215

[B9] Rocha G. S. Freire M. A. M. Brimblecombe K. R. (2026). The basal ganglia upside down: noncanonical direct and indirect pathways emerging from striosomes modulate dopamine release and motor behavior. J. Neurophysiol. 135, 1279–1285. doi: 10.1152/jn.00527.2025 42012094

[B10] Varin C. Cornil A. Houtteman D. Bonnavion P. de Kerchove d'Exaerde A. (2023). The respective activation and silencing of striatal direct and indirect pathway neurons support behavior encoding. Nat. Commun. 14:4982. doi: 10.1038/s41467-023-40677-0 37591838 PMC10435545

